# Exercise-induced vitamin D receptor and androgen receptor mediate inhibition of IL-6 and STAT3 in muscle

**DOI:** 10.1016/j.bbrep.2023.101621

**Published:** 2023-12-21

**Authors:** Seiji Hashimoto, Tatsuya Hosoi, Mitsutaka Yakabe, Shoya Matsumoto, Masayoshi Hashimoto, Masahiro Akishita, Sumito Ogawa

**Affiliations:** a Department of Geriatric Medicine, Graduate School of Medicine, The University of Tokyo, 7-3-1 Hongo, Bunkyo-ku, Tokyo, 113-8655, Japan; b Department of General Medicine, Graduate School of Medicine and Dental Sciences, Tokyo Medical and Dental University, 1-5-45 Yushima, Bunkyo-ku, Tokyo, 113-8519, Japan

**Keywords:** Vitamin D receptor, Androgen receptor, Interleukin-6, STAT3

## Abstract

**Background:**

Skeletal muscle produces interleukin-6 (IL-6) during exercise as a myokine. Although IL-6 is required for skeletal muscle regeneration, its action increases the expression of myostatin and other proteins involved in muscle atrophy, resulting in skeletal muscle atrophy. In this study, we clarified the effects exercise-induced vitamin D receptor (VDR) and androgen receptor (AR) expression on IL-6 and signal transducer and activator of transcription 3 (STAT3) *in vivo* and *in vitro.*

**Method:**

C2C12 myotubes were subjected to electric pulse stimulation (EPS) *in vitro*. To evaluate VDR and AR function, a VDR/AR agonist and antagonist were administered before EPS to C2C12 myotubes. C57BL6 mice underwent 4 weeks of exercise. The expression levels of proteolytic-associated genes, including CCAAT/enhancer-binding protein delta (C/EBPδ) and myostatin, were measured by quantitative real-time polymerase chain reaction, and phosphorylated and total STAT3 levels were measured by Western blot analysis.

**Result:**

The expression of VDR and AR mRNA was induced following EPS in C2C12 myotubes. IL-6 mRNA expression was also increased with a peak at 6 h after EPS and p-STAT3/STAT3 ratio reciprocally decreased. Although VDR/AR agonist administration decreased IL-6 mRNA expression and p-STAT3/STAT3 ratio, these two endpoints increased after treatment with VDR/AR antagonist, respectively. Exercise in mice also increased the expression of VDR/AR and IL-6 mRNA and decreased p-STAT3/STAT3 ratio.

**Conclusion:**

Exercise-induced VDR and AR expression results in the suppression of IL-6 mRNA and STAT3 phosphorylation in skeletal muscle.

## Highlights

• EPS stimulation and exercise increased IL-6 mRNA expression in C2C12 myotubes and skeletal muscle, and decreased the ratio of phosphorylated STAT3.
• Administration of VDR/AR antagonists increase IL-6 mRNA expression and STAT3 phosphorylation ratio, whereas administration of VDR/AR agonists has opposite effect.
• Exercise-induced VDR/AR might regulate the activation of STAT3.

## 1 Introduction

Sarcopenia is a pathological condition characterized by a decrease in skeletal muscle mass and strength. Primary sarcopenia is defined as having no obvious cause other than aging [1]. After the age of 50, muscle mass decreases by 0.80–0.98 % annually in men and 0.64–0.70 % annually in women [2]. As skeletal muscle mass and strength decline with age, the risk of falls and the requirement for nursing care increases. Therefore, interventions for sarcopenia are considered crucial [3]. Although there is no established treatment for sarcopenia, some pharmacological approaches, such as vitamin D and testosterone as well as nonpharmacological approaches, such as nutrition and exercise have been proposed [4]. Vitamin D and testosterone are thought to act via the nuclear vitamin D receptor (VDR) and androgen receptor (AR), respectively. Moreover, vitamin D and testosterone affect anabolic and catabolic pathways in skeletal muscle and are associated with mitochondrial function, which results in a protective effect in skeletal muscle [5,6]. The expression VDR and AR in skeletal muscle decreases with age [7,8], suggesting that vitamin D and androgen effects decrease in the elderly. The relationship between exercise and VDR/AR activity in skeletal muscle is not well understood, and there are no reports focused on the properties of both VDR and AR. Exercise has multiple effects and is considered effective in preventing sarcopenia and important for healthy aging. One of the underlying factors is myokines produced by skeletal muscle during exercise [9]. Myokine production was induced not only by exercise *in vivo*, but also by electric pulse stimulation (EPS), an *in vitro* exercise model [10]. Various myokines, such as interleukin 6 (IL-6), brain-derived neurotropic factor, and irisin, are produced by skeletal muscle [11]. Of these, IL-6 is produced by various cells, such as immune-mediated cells and fibroblasts [12], which not only act as an inflammatory cytokine, but also as a myokine. IL-6 stimulates cell proliferation and promotes muscle differentiation through the expression of MyoD and myogenin in C2C12 cells [13]. IL-6 also promotes protein synthesis through the rapamycin complex 1 (mTORC1) [14]. These results suggest that IL-6 may be involved in muscle differentiation and its synthesis. In contrast, IL-6 may also be associated with muscle atrophy-related genes [myostatin, muscle atrophy F-box protein (MAFbx)/Atrogin-1, muscle RING finger 1 (MuRF-1)] via signal transducer and activator of transcription 3 (STAT3) [15,16]. Moreover, IL-6 expression level was elevated in disuse model mice and administration of an IL-6 inhibitor to the mice reduced muscle atrophy-related gene expression [17]. With respect to its effect on IL-6, vitamin D and testosterone decrease IL-6 expression [18,19]. Although these previous studies suggest a relationship between vitamin D/androgen action and the IL-6/STAT3 signaling pathway, little is known about the change in VDR and AR expression and its function during exercise. In this study, we determined the effect of exercise-induced VDR and AR expression on IL-6 and STAT3 *in vivo* and *in vitro.*

## 2 Materials and methods

### 2.1 Regents

Dulbecco's modified essential medium (DMEM) was purchased from FUJIFILM Wako pure chemical corporation (Osaka, Japan), fetal bovine serum (FBS) from Biowest (Nuaillé, France), horse serum and penicillin-streptomycin from Thermo Fisher Scientific (Waltham, MA, USA). C2C12 cells were purchased from the American Type Culture Collection (Manassas, VA, USA). Calcitriol and testosterone were purchased from FUJIFILM Wako pure chemical corporation, MeTC7 from MedchemExpress (NJ, USA), flutamide from Sigma-Aldrich (St. Louis, USA)

### 2.2 Cell culture

C2C12 cells, a murine myoblast cell line, were cultured in DMEM added in 10 % FBS, 100 U/mL penicillin, and 100 μg/mL streptomycin under 5 % CO_2_ at 37 °C. Differentiation was induced by changing the medium (DMEM added in 2 % horse serum) after the cells reached 80–90 % confluent. The differentiation medium was changed every 2 days. C2C12 cells were cultured in differentiation medium for 5 days.

### 2.3 Electric pulse stimulation

C-Dish (IonOptix, MA, USA) was placed in a 6 well plate and electrically stimulated with C-Pace pulse generator (IonOptix, MA, USA). EPS was applied as a 2 ms duration at 11.5 V and 1 Hz. EPS conditions were based on previous report [20]. Flutamide (10 μM) and MeTC7 (10 μM) were pretreated 30 min before EPS. Calcitriol (100 nM) and testosterone (10 nM) were pretreated 16 h before EPS. These reagent conditions were also based on previous reports [[21], [22], [23], [24]].

### 2.4 Animals

Male C57BL/6J mice were purchased from Nippon CLEA (Tokyo, Japan). They were housed individually and were maintained in a controlled environment with a 12 h:12 h light:dark cycle. The mice were provided *ad libitum* access to standard chow and water. They were acclimated for 2 weeks and were used for the experiments at 11 weeks of age.

Exercise was performed 5 days a week for 4 weeks based on the previous report [25]. The climbing procedures used 3 sets/day and 4 repetitions/set, with 1min of rest between sets. In the first week, we subjected the mice to 5 % body weight (BW) weight-loading, then for the 2–4 weeks phase they were loaded with 25 %, 50 % and 75 % BW, respectively. This study was approved by the Animal Care and Use Committee of the University of Tokyo.

### 2.5 Western blotting and antibodies

C2C12 myotubes were lysed in radioimmunoprecipitation assay buffer combined with a protease inhibitor cocktail (cOmplete Mini, Roche Applied Science, Penzberg, Germany) and a phosphatase inhibitor cocktail (PhosSTOP, Roche Applied Science). Mice muscle tissue was immersed in T-PER (Tissue Protein Extraction Reagent, Thermo Fisher Scientific) with cOmplete Mini and PhosSTOP, and pulverized using a Cell Destroyer (Bio Medical Science Inc, Tokyo, Japanmai). The proteins in these lysates were separated using SDS-PAGE and were electro-transferred onto a polyvinylidene difluoride membrane. We used 4 μg of protein for each Western blot. The membranes were blocked using Blocking One (Nacalai Tesque Inc, Kyoto, Japan) and probed using appropriate primary and secondary antibodies (Anti-phospho STAT3 (CST #9145, 1:2000), anti-STAT3 (CST #4904, 1:2000)). After immunoblotting, the proteins were visualized using an ECL Prime Western Blotting Detection Regent (Cytiva, Tokyo, Japan). The chemiluminescence images were scanned using a LuminoGraph I (Atto Corp, Tokyo, Japan). After testing for phosphoSTAT3, we stripped and reprobed membranes. Blots were quantified using Image J software (National Institutes of Health, MD, USA).

### 2.6 Quantitative real-time PCR analysis

RNA was extracted from C2C12 myotubes, using a QIAshredder and RNeasy Mini Kit (Qiagen, Dusseldorf, Germany). Harvested gastrocnemius muscles were immersed in RNAlater (Qiagen, Dusseldorf, Germany) to stabilize RNA in tissues. RNA extraction was performed using a RNeasy Fibrous Tissue Mini Kit (Qiagen, Dusseldorf, Germany). DNase I treatment was performed using an RNase-Free DNase Set (Qiagen, Dusseldorf, Germany). The cDNA was synthesized from the RNA samples using a ReverTra Ace qPCR RT Kit (Toyobo, Osaka, Japan) and used according to the manufacturer's instructions. The cDNA was mixed with SYBR Green Master Mix (Applied Biosystems, CA, USA) and specific primers for each gene, and then subjected to quantitative real-time PCR using a StepOnePlus Real-Time PCR System (Applied Biosystems). Data were normalized using glyceraldehyde-3-phosphate dehydrogenase (GAPDH) for each sample and calculated using the comparative critical threshold (Ct) method. The sequences of PCR primers are provided in Table 1.

**Table 1:** Primer list.

Gene	Forward (5′-3′)	Reverse (5′-3′)
GAPDH	AGGTCGGTGTGAACGGATTTG	GGGGTCGTTGATGGCAACA
VDR	GAATGTGCCTCGGATCTGTGG	GGTCATAGCGTTGAAGTGGAA
AR	AAGTGAATGGTCTTGGC	TTACAGGTCTGGTGCAAGCC
IL-6	CAATGCTCTCCTAACAGATAAG	AGGCATAACGCACTAGGT
Myostatin	CTCCAGAATAGAAGCCATA	GCAGAAGTTGTCTTATAGC
C/EBPδ	CTCCAGGGTCTAAATACATAGC	CTCACAGCAGTCCACAAG

### 2.7 Statistical analysis

All data are presented as the mean ± standard error of the mean (SEM). Comparisons between two samples were conducted using Student's t-test. Comparisons between three or more samples were conducted using one-way analysis of variance (ANOVA). Probability values of *p* < 0.05 were considered statistically significant.

## 3 Results

### 3.1 EPS increases VDR and AR expression *in vitro*

**Fig. 1:**
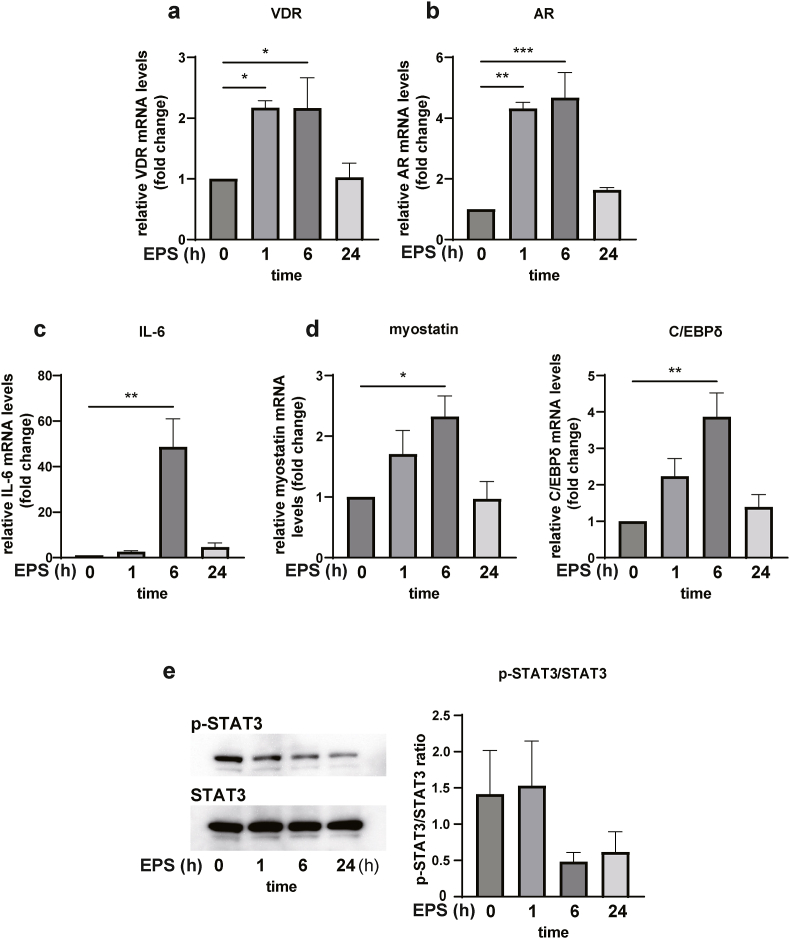
EPS-induced expression of VDR. AR, IL-6, myostatin, C/EBPδ mRNA and STAT3 phosphorylation in differentiated C2C12 cells. a–d) Comparison of mRNA levels at each EPS time point. e) Immunoblotting and quantitation of p-STAT3 and STAT3 protein levels at each EPS time. Data are presented as the mean ± SEM. *p ＜ 0.05, **p ＜ 0.01. ***p ＜ 0.001; ns: not significant.

Using C2C12 myotubes, EPS was applied for 24 h under the following conditions: 1 Hz, 2 ms, and 11.5 V/35 mm. The expression of VDR peaked at 6 h following EPS stimulation, which was similar to AR (Fig. 1a and b). Next, we determined the expression of IL-6/STAT3 and muscle atrophy-related genes at each time point following EPS. EPS significantly increased IL-6 mRNA expression after 6 h (Fig. 1c). Myostatin and C/EBPδ mRNA were also increased after 6 h of EPS stimulation (Fig. 1d). In contrast, p-STAT3/STAT3 ratio was decreased in response to EPS stimulation (Fig. 1e).

### 3.2 Vitamin D and androgen signaling downregulates IL-6 and STAT3

**Fig. 2:**
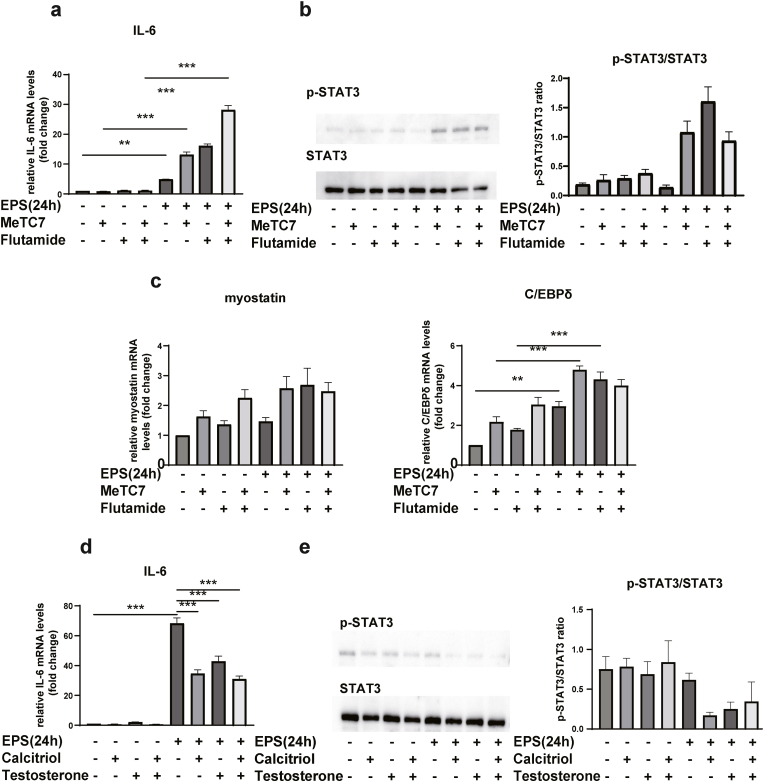
Expression changes of IL-6, myostatin, C/EBPδ mRNA and STAT3 phosphorylation by preadministration of VDR/AR agonists and antagonists with EPS, in differentiated C2C12 cells. a,c,d) The expression of each mRNA was compared. b,e) Immunoblotting and quantitation of p-STAT3 and STAT3 levels. Data are presented as the mean ± SEM. *p ＜ 0.05, **p ＜ 0.01. ***p ＜ 0.001; ns: not significant.

Sustained effects of IL-6 are thought to be associated with muscle atrophy-related genes; thus, we examined their expression after 24 h of EPS stimulation. The VDR antagonist MeTC7, and AR antagonist flutamide were administered before EPS. Administration of each inhibitor markedly increased IL-6 expression compared with EPS alone (Fig. 2a). Furthermore, p-STAT3/STAT3 ratio as well as the expression of myostatin and C/EBPδ mRNA were also increased (Fig. 2b and c). Conversely, preadministration of vitamin D and testosterone decreased IL-6 mRNA expression, whereas p-STAT3/STAT3 ratio was also decreased compared with the control (Fig. 2d and e).

### 3.3 Exercise increases VDR and AR expression *in vivo*

**Fig. 3:**
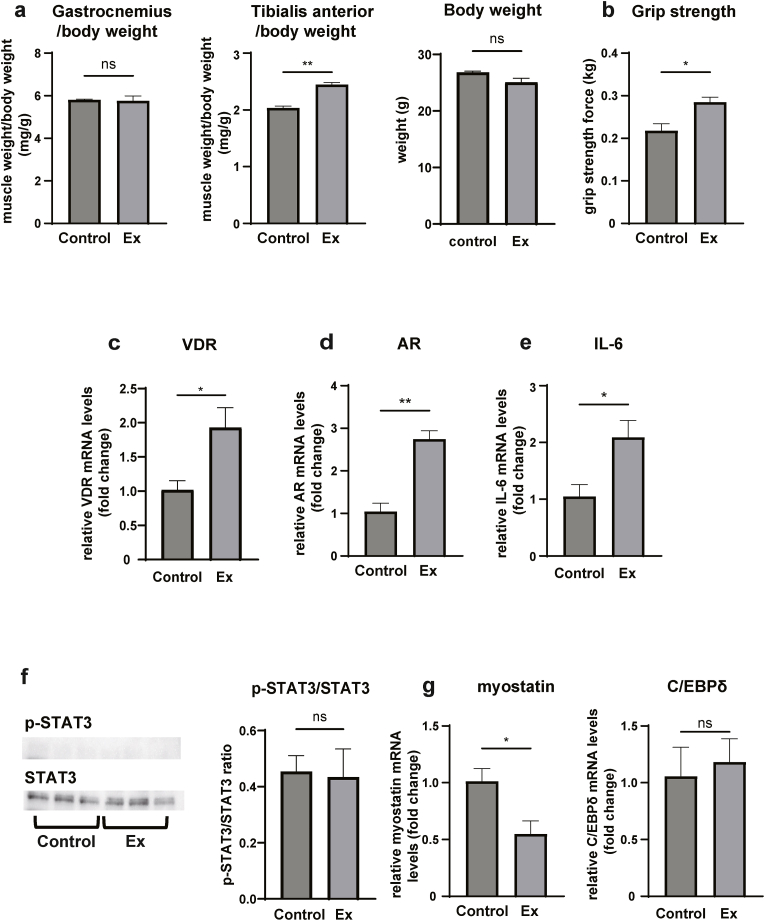
Downregulation of IL-6/STAT3 signaling pathway in exercised mice. a) The muscle weight/body weight ratio of control and exercise group (Ex) are shown. b) Grip strength of extremities. c–e, g) The expression of each mRNA. f) Immunoblotting and quantitation of p-STAT3 and STAT3 levels. Data are presented as the mean ± SEM. *p ＜ 0.05, **p ＜ 0.01. ***p ＜ 0.001; ns: not significant.

**Fig. 4:**
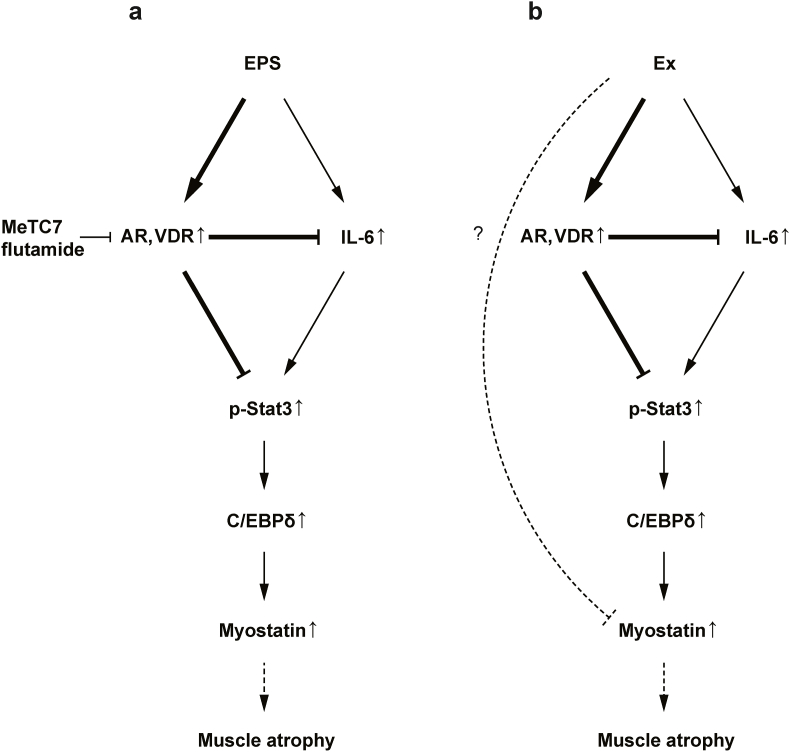
Schematic diagram of the relationship between VDR/AR and IL-6/STAT3 signaling in skeletal muscle. The expression of VDR and AR is induced by EPS and exercise, respectively and STAT3 is suppressed via induced VDR and AR.

C57BL6 mice were subjected to 4 weeks of exercise and the gastrocnemius and tibialis anterior muscles were isolated. There was no significant difference in gastrocnemius muscle weight; however, the tibialis anterior muscle weight increased in the exercise group. Moreover, grip strength was also significantly increased in the exercise group (Fig. 3a and b). Next, we examined the expression of VDR and AR in the gastrocnemius muscle and found that expression of both receptor mRNAs was elevated (Fig. 3c and d). The expression of IL-6 mRNA was also increased in the exercise group, whereas p-STAT3/STAT3 ratio was not increased (Fig. 3e and f). These results were similar to those observed *in vitro*. In the exercise group, while myostatin mRNA was decreased, no significant difference was observed in C/EBPδ mRNA expression (Fig. 3g). Based on these results, it was revealed and suggested that while IL-6 expression increases during exercise, exercise-induced VDR and AR suppresses STAT3 phosphorylation (Fig. 4).

## 4 Discussion

In this study, EPS and exercise increased the simultaneous expression of VDR and AR. Our findings also suggest that VDR might prevent muscle atrophy similar to the involvement of AR on skeletal muscle. Furthermore, we found that STAT3 activation was suppressed by EPS and exercise, and VDR and AR were also involved in this process. Based on our *in vitro* analysis, the expression of C/EBPδ was increased despite decreased STAT3 phosphorylation (Fig. 1d and e). This may be the result of NF-kB pathway activation, because it was reported that EPS activates the NF-kB signaling pathway and C/EBPδ is induced via NF-kB [26,27].

We also demonstrated that during exercise, VDR inhibited STAT3 activation in an IL-6-independent manner. Our results are consistent with previous reports showing activation of STAT3 in the skeletal muscle of VDR knockout mice [28]. Our current findings also revealed that pharmacological inhibition of VDR and AR increased IL-6 expression and STAT3 phosphorylation (Fig. 2a and b). EPS-induced VDR and AR expression decreased p-STAT3/STAT3 ratio compared with a VDR and AR agonist alone (Fig. 2d and e). Therefore, the induction of VDR and AR expression might be important for the regulation of IL-6 and STAT3. Further studies are needed to clarify which of the ligand or the receptor is more important for the regulation of muscle properties, because exercise is known to increase the expression of CYP27B1, and vitamin D synthase, as well as the production of testosterone and 5α-reductase [21,29]. Additionally, IL-6 expression was increased by coadministration of VDR and AR inhibitors compared with each administered alone (Fig. 2a). As vitamin D and testosterone are known to act through different specific receptors, vitamin D and testosterone might exhibit additive or synergetic effects.

Based on our *in vivo* analysis, the expression of myostatin was decreased despite no significant change of C/EBPδ (Fig. 3g). Further studies might reveal alternative myostatin regulatory pathways. Expression of VDR and AR are decreased in the older population compared with younger subjects [7,8], further suggesting that the inability to suppress STAT3 might induce muscle atrophy in older individuals. Furthermore, certain types of exercise, which do not induce VDR or AR expression, might not prevent the expression of muscle atrophy-related genes. Future studies may determine more in detail which type of exercise regulates VDR and AR expression and its related genes.

In summary, our study suggests that VDR and AR expression during exercise might regulate IL-6/STAT3 signaling mediated by the suppression of STAT3 phosphorylation. In practice, it would be expected that an ideal exercise program for the elderly might be developed in the future. Identification of the underlying molecular mechanisms responsible for the effects of exercise, vitamin D, and androgens might lead to the discovery of treatments for age-related sarcopenia.

## Funding sources

This work was supported by Grant-in-Aid for Scientific Research from the 10.13039/501100001691Japan Society for the Promotion of Science (22K07465) and by AMED (22675555).

## Data statement

The data are available from the corresponding author upon reasonable request.

## CRediT authorship contribution statement

**Seiji Hashimoto:** Writing – original draft, Visualization, Investigation, Formal analysis. **Tatsuya Hosoi:** Writing – review & editing, Validation, Resources, Investigation. **Mitsutaka Yakabe:** Writing – review & editing, Validation, Investigation. **Shoya Matsumoto:** Investigation. **Masayoshi Hashimoto:** Supervision. **Masahiro Akishita:** Writing – review & editing, Supervision. **Sumito Ogawa:** Writing – review & editing, Writing – original draft, Supervision, Project administration, Methodology, Funding acquisition, Conceptualization.

## Declaration of competing interest

The authors declare that they have no known competing financial interests or personal relationships that could have appeared to influence the work reported in this paper.
