# Supplementary material for: Exercise-induced vitamin D receptor and androgen receptor mediate inhibition of IL-6 and STAT3 in muscle
Source: Biochem Biophys Rep. 2023 Dec 21;37:101621. doi: 10.1016/j.bbrep.2023.101621 (PMC10776921; doi:10.1016/j.bbrep.2023.101621)
Supplement: Multimedia component 1 [file mmc1.pptx]

## Slide 1
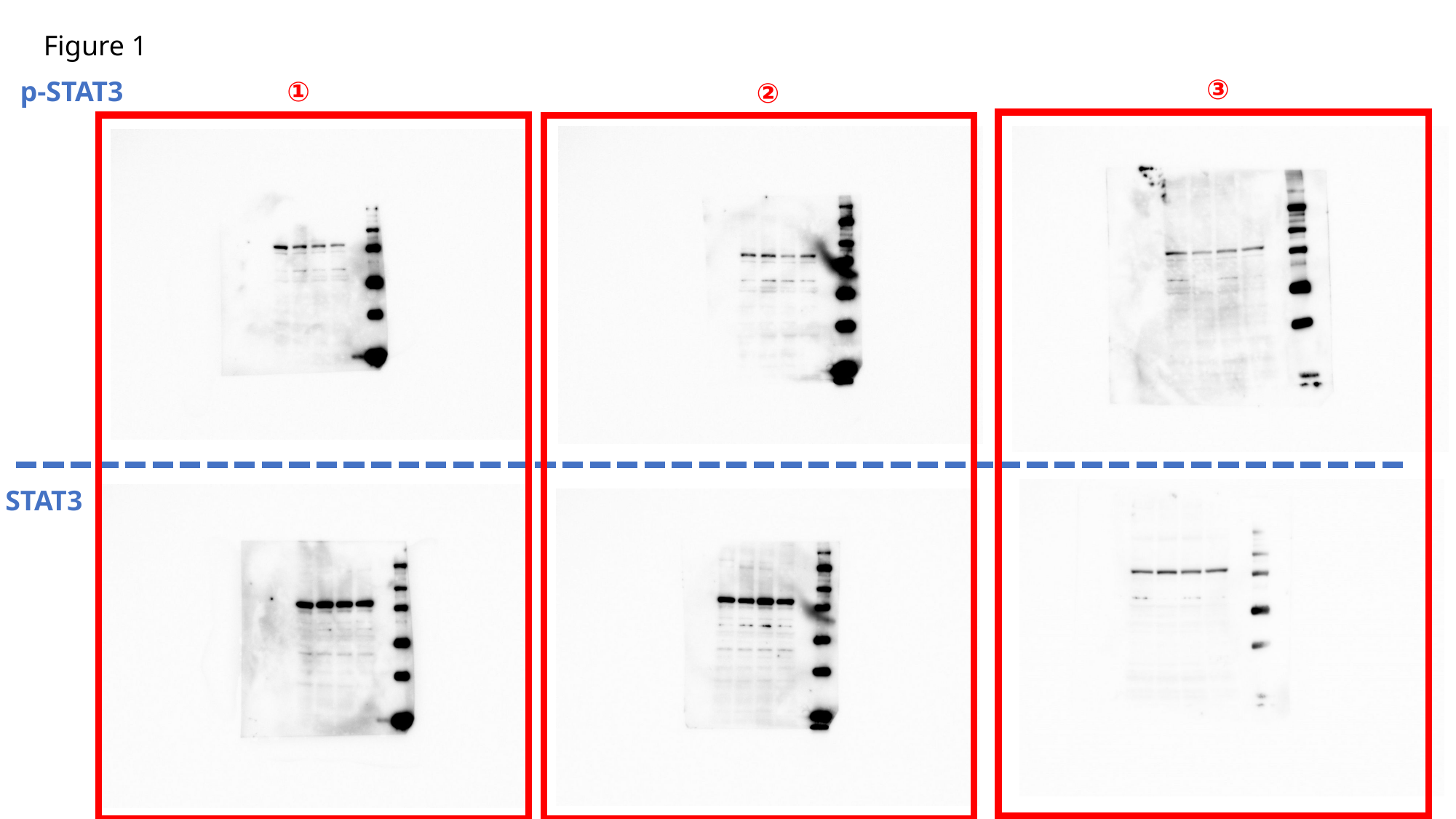

Figure 1
③
p-STAT3
①
②
STAT3

## Slide 2
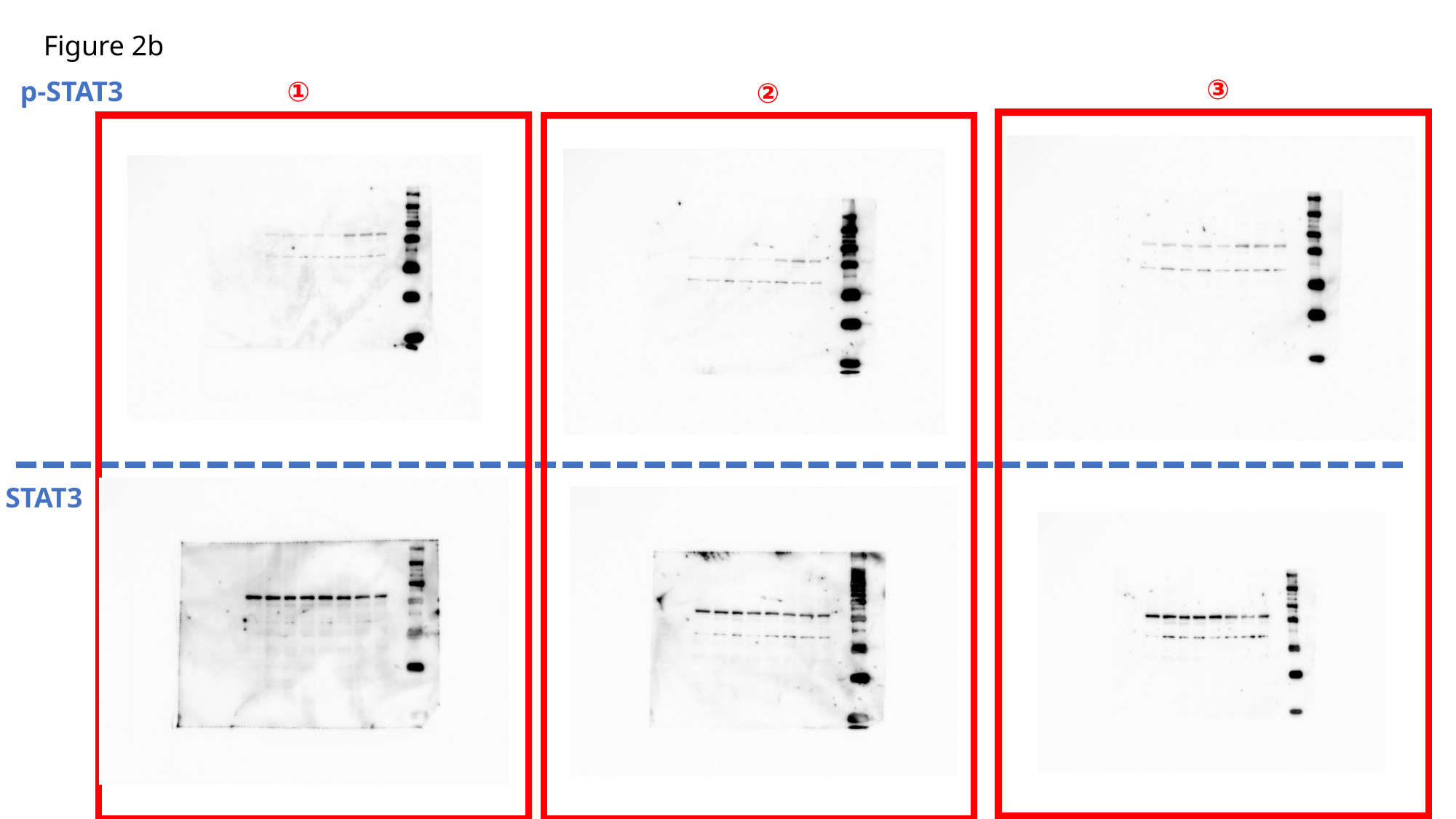

Figure 2b
③
p-STAT3
①
②
STAT3

## Slide 3
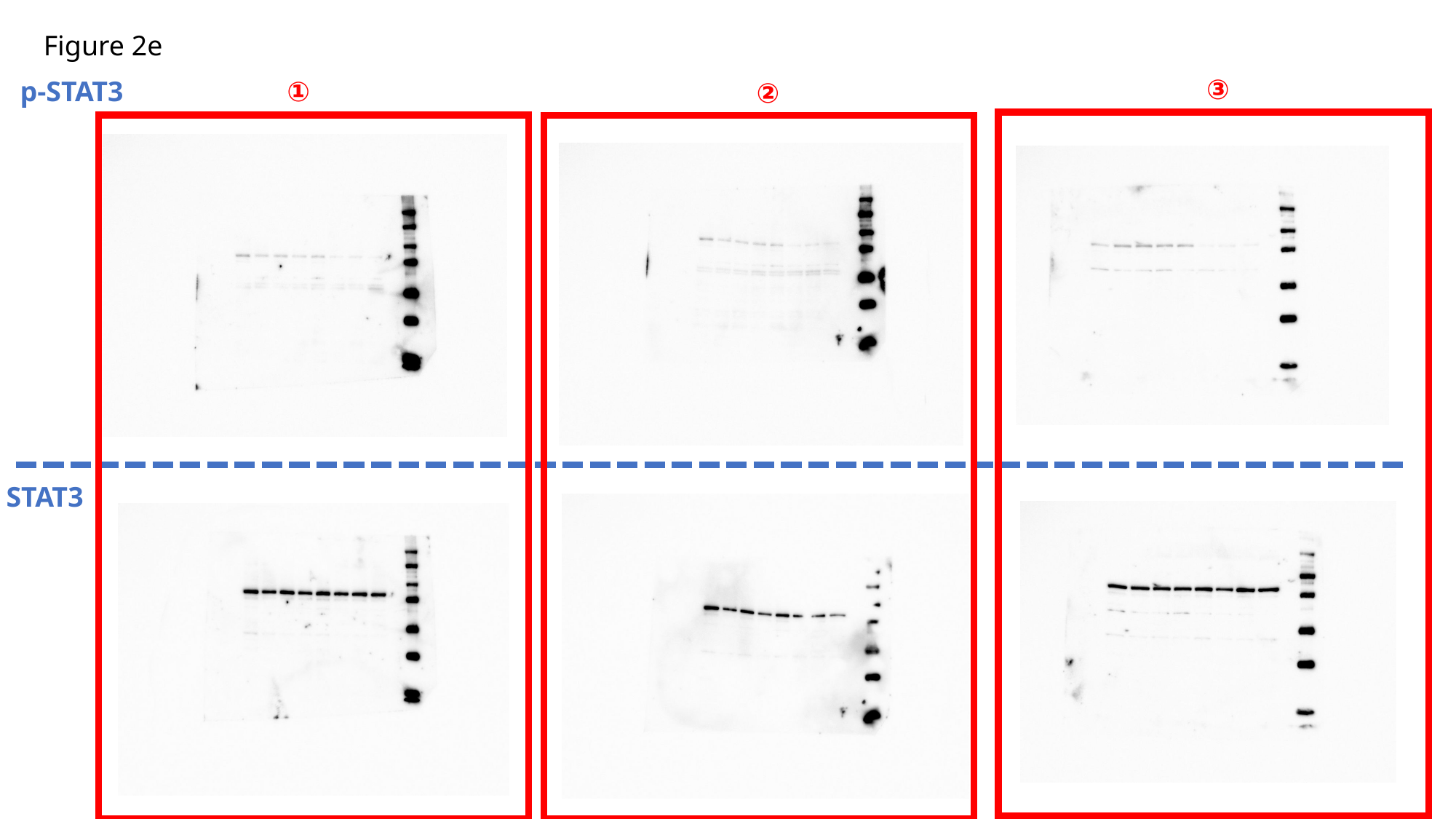

Figure 2e
③
p-STAT3
①
②
STAT3

## Slide 4
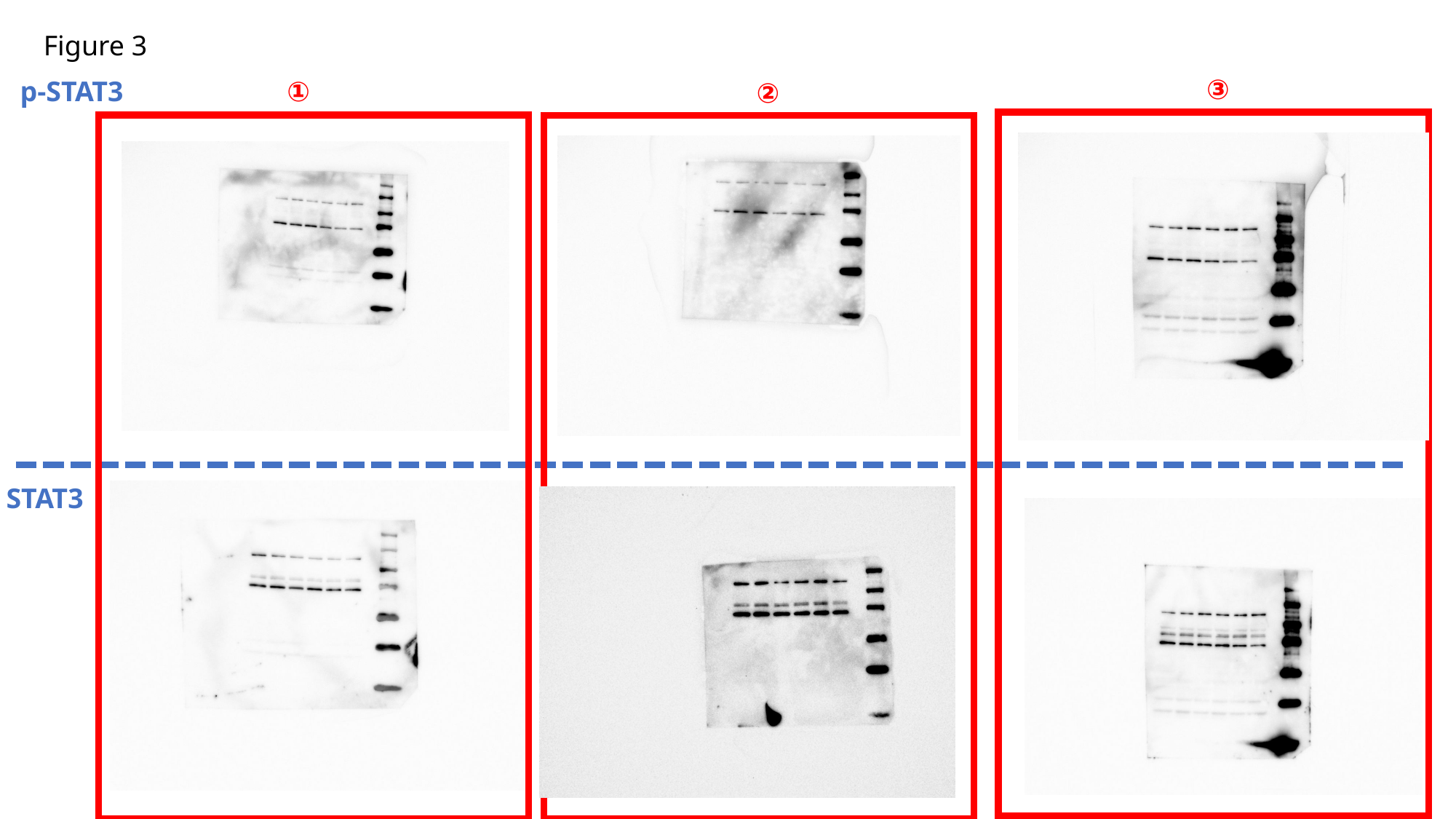

Figure 3
③
p-STAT3
①
②
STAT3
